# Successful anesthetic management for total mastectomy in a pregnant woman using general anesthesia combined with continuous erector spinae plane block: a case report

**DOI:** 10.1186/s40981-019-0245-y

**Published:** 2019-03-18

**Authors:** Atsushi Sawada, Sayaka Sotome, Mikako Kusakai, Michiaki Yamakage

**Affiliations:** Department of Anesthesiology, Sapporo Medical University School of Medicine, South 1, West 16, Chuo-ku, Sapporo, Japan

**Keywords:** Pregnant woman, Mastectomy, Erector spinae plane block

## Abstract

**Background:**

Anesthetic considerations for surgery during pregnancy include the safety of both mother and fetus. We successfully administered anesthesia for total mastectomy to a pregnant woman using general anesthesia combined with continuous erector spinae plane block.

**Case presentation:**

A 41-year-old woman was scheduled to undergo total mastectomy at 18 weeks’ gestation. Hence, we decided to administer general anesthesia combined with continuous erector spinae plane block to minimize physiological stress on both mother and fetus. Continuous erector spinae plane block provided sufficient postoperative analgesia for our patient, completely eliminating the need for additional rescue analgesia during the entire postoperative period.

**Conclusions:**

General anesthesia combined with continuous erector spinae plane block provided adequate analgesia without maternal hypotension in a pregnant woman undergoing total mastectomy.

## Background

Breast cancer is one of the most common cancers in pregnant women [1]. Anesthetic considerations for surgery during pregnancy include the safety of both mother and fetus. The fetus might be at risk of intraoperative hypoxia secondary to maternal hypotension. Further, surgical procedures and postoperative pain increase the potential risks of miscarriage, preterm delivery, and threatened premature labor [2]. Hence, the use of regional block techniques that have minimal effects on maternal hemodynamics might be a safer option during surgery in pregnant women.

Cutaneous innervation of the breast is mainly derived from the intercostal nerves, with a small contribution from the supraclavicular nerves [3]. Among the various nerve block techniques used during thoracic surgery, the erector spinae plane (ESP) block is a relatively new truncal nerve block first described for thoracic analgesia [4]. A cadaveric study revealed that ESP block leads to the spread of the local analgesic into a wide range of intercostal spaces from a single point of injection [5]. Here, we report a case of successful anesthetic management for total mastectomy in a pregnant woman using general anesthesia combined with continuous ESP block.

To the best of our knowledge, this is the first report discussing the efficacy of continuous ESP block for mastectomy in a pregnant woman.

## Case presentation

A 41-year-old woman (weighing 68 kg, 157 cm tall) with a previous history of childhood asthma became pregnant during follow-up of breast cancer. She was scheduled to undergo total mastectomy with sentinel lymph node biopsy at 18 weeks of gestation. In order to minimize physiological stress on both mother and fetus during the perioperative period, we decided to administer general anesthesia combined with continuous ESP block.

Her physical examination and preoperative laboratory tests indicated no abnormalities. After establishing standard monitoring, including bispectral index (BIS) and neuromuscular monitoring, general anesthesia was induced with intravenous propofol (target-controlled infusion [TCI] of 3.3 μg/mL) and remifentanil (0.25 μg/kg/min). Following calibration of the train-of-four (TOF) monitor, we administered rocuronium at fractional doses of 10 mg until the required degree of muscle relaxation was achieved. Tracheal intubation was uneventfully performed.

**Fig. 1:**
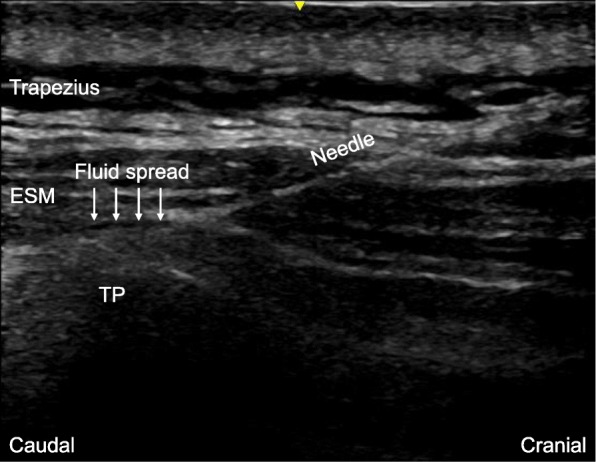
Ultrasound image of erector spinae plane block. The end point is the visible spread of the local anesthetic fluid, resulting in the lifting of the erector spinae muscles. ESM erector spinae muscles, TP transverse process

After placing the patient in the right lateral position, a high-frequency linear ultrasound transducer (LOGIQe, GE Healthcare, Wauwatosa, Wisconsin) was aseptically placed on the patient’s back in a longitudinal parasagittal orientation approximately 3 cm from the midline. The erector spinae muscles were identified superficial to the tip of the T3 transverse process. An 18-gauge, 100-mm needle (Contiplex® S Ultra, B. Braun, Melsungen, Germany) was inserted using an in-plane approach and in a cranial-to-caudal direction to contact the tip of the T3 transverse process (Fig. 1). The location of the needle tip was confirmed by visible fluid spread resulting in the lifting of the erector spinae muscles. A total of 20 mL of 0.25% levobupivacaine was injected through the needle, followed by insertion of a 19-gauge catheter 5 cm beyond the needle tip. Then, an infusion of 0.25% levobupivacaine into the erector spinae plane was started at the rate of 6 mL/h. General anesthesia was maintained with propofol (TCI of 3.0–3.3 μg/mL) to keep the BIS between 40 and 60, remifentanil (0.05–0.1 μg/kg/min) to maintain systolic blood pressure within 20% of preoperative values, and intermittent administration of 10 mg rocuronium to keep TOF counts below 2. The operative time was 111 min. The patient’s hemodynamics remained stable during the surgery. One thousand milligrams of acetaminophen was intravenously administered 15 min before the end of surgery. After full recovery from general anesthesia, the patient was extubated and transferred to the general ward of the department of surgical oncology.

Postoperative pain was measured using a numerical rating scale (NRS; an 11-point scale, 0 was no pain and 10 was the worst pain imaginable) immediately after recovery from general anesthesia and at varied intervals postoperatively (at 2, 6, 24, and 36 h). NRS scores remained at 1 point throughout the postoperative period, indicating minimal pain. At 2 h after surgery, postoperative assessment of the fetus with Doppler ultrasound revealed no abnormalities. The catheter of the ESP block was removed at 24 h after surgery. Postoperative pain did not worsen after termination of the block. The patient did not need any additional analgesics during the postoperative period and was discharged home on the 15th day after surgery with no adverse effects on either mother or fetus.

## Conclusions

Thoracic epidural anesthesia is associated with the potential risks of accidental dural puncture, subarachnoid injection, and maternal hypotension, which would adversely affect uteroplacental blood flow [6]. Although the paravertebral block is a useful regional anesthetic for breast surgery, the technique is complicated with the potential risk of pneumothorax. It was previously reported that ESP block provides complete surgical anesthesia for radical mastectomy [7]. We performed ESP block for breast cancer surgery in our pregnant patient because it is associated with a lower risk of complications than thoracic epidural anesthesia and paravertebral block. Although two cadaveric studies were previously reported regarding the spread of local anesthetic drugs to the paravertebral space following ESP block, they have led each contradictory conclusion [5, 8]. Hence, whether or not ESP block can block thoracic sympathetic nerves remains unknown, ESP block provided our pregnant patient with adequate analgesia for breast cancer surgery without maternal hypotension during the surgery.

Management of postoperative analgesia in pregnant women requires specific considerations because non-steroidal anti-inflammatory drugs, one of the most common analgesics, are generally avoided. Like bupivacaine, levobupivacaine, which is the S-enantiomer of bupivacaine, is safe for use in pregnant women [9], with less cardiac and central nervous system toxicity [10]. In our patient, continuous ESP block at the rate of 6 mL/h of 0.25% levobupivacaine provided adequate and safe postoperative analgesia, eliminating the need for rescue analgesia during the entire postoperative period. Although we did not measure the area of the sensory block in the postoperative period, we expected that the continuous ESP block provided sensory block in the T2 to T6 dermatomes, which is the area affected by the surgical invasiveness of total mastectomy.

In summary, general anesthesia combined with continuous ESPB provided adequate analgesia without maternal hypotension in our pregnant patient undergoing total mastectomy.

## Abbreviations

BIS: Bispectral index
ESPB: Erector spinae plane block
NRS: Numerical rating scale
TCI: Target-controlled infusion
TOF: Train-of-four
